# Molecular Insights into the Interaction between CD147 and the SARS-CoV‑2 Spike Protein

**DOI:** 10.1021/acsomega.5c03562

**Published:** 2025-08-05

**Authors:** Milton S. Gonzalez-Serrano, Li-Yu Chen, Hemi Desai, Thi Huynh Ho, Doris Heinrich, Thuat Thanh Trinh, Thi-Huong Nguyen

**Affiliations:** ∇ Institute for Bioprocessing and Analytical Measurement Techniques, Heiligenstadt 37308, Germany; ‡ Department of Chemical and Biomolecular Engineering, The Ohio State University, Columbus, Ohio 43210-1132, United States; § Hochschule Anhalt University of Applied Sciences, Köthen 06366, Germany; ∥ Laboratory for Computational Physics, Institute for Computational Science and Artificial Intelligence, Van Lang University, Ho Chi Minh City 700000, Vietnam; ⊥ Faculty of Mechanical - Electrical and Computer Engineering, School of Technology, Van Lang University, Ho Chi Minh City 700000, Vietnam; # Faculty of Mathematics and Natural Sciences, Technische Universität Ilmenau, 98694 Ilmenau, Germany; 7 Porelab, Department of Chemistry, Norwegian University of Science and Technology, Høgskoleringen 5, Trondheim 7491, Norway

## Abstract

Cluster of differentiation 147 (CD147), a transmembrane glycoprotein, has been identified as a potential auxiliary receptor for the SARS-CoV-2 spike protein (SP), contributing to COVID-19 infection. However, the detailed binding characteristics of this interaction remain unclear. Here, we characterized SP-CD147 binding using Enzyme-Linked Immunosorbent Assay (ELISA) and single-molecule force spectroscopy (SMFS) under varying contact times and temperatures. While SP binds CD147 with lower force than angiotensin-converting enzyme 2 (ACE2) at normal body temperatures, its binding strength to CD147 significantly increases under fever-like conditions, contrasting with SP-ACE2 interactions, which weaken at elevated temperatures. We further investigated the role of heparin in modulating this interaction and found that heparin independently binds both CD147 and SP, enhancing the interaction force between CD147 and SP, as confirmed by Quartz Crystal Microbalance (QCM) and SMFS. Molecular dynamics simulations and binding free energy calculations provided atomic-level insights into the stabilizing role of heparin within the SP/CD147 complex. These findings offer a comprehensive characterization of the CD147-SP interaction, revealing potential therapeutic avenues for COVID-19 intervention.

## Graphical abstract

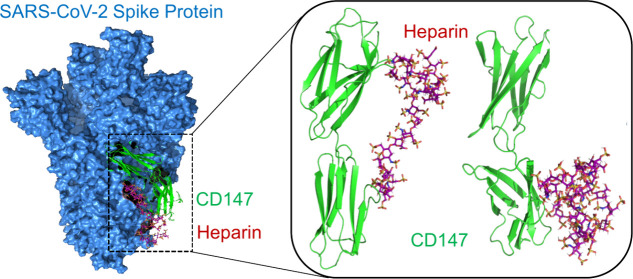

## Introduction

As of January 21, 2025, the COVID-19 pandemic has resulted in over 7 million confirmed deaths worldwide.^1^ Despite vaccines and treatments being widely available, the virus’s high mutation rate remains a significant challenge, posing ongoing risks, especially to the elderly and immunocompromised individuals.^2^

Even though the angiotensin-converting enzyme 2 (ACE2) has been established as the primary receptor for viral entry into host cells,^3^ other biological targets have been identified as potential sites for infection.^2^ The transmembrane glycoprotein cluster of differentiation 147 (CD147) has recently been found to interact with the viral SARS-CoV-2 spike protein (SP) recognition binding domain.^2^ CD147 is also known as EMMPRIN or basiginare expressed on multiple cell types, including epithelial, immune, tumor, vascular endothelial cells, and myeloid cells, and it is involved in other inflammatory responses.^4^ The CD147 gene has an open reading frame that encodes 269 amino acids in the coding region, including two C2-type immunoglobulin regions in the extracellular N-terminal sequence, 24 amino acids within the transmembrane region, and 39 amino acids within the C-terminal intracellular region.^5^ The molecular weight of CD147 varies according to its glycosylation status. There are two hypoxia-inducible factor binding sites in the 3′-flanking region of the CD147 gene.^6^ Interestingly, CD147 is an overexpressed biomarker in cancerous tissues, making it an attractive target for cancer treatment,^7,8^ CD147 has multiple functions in different cells and different stages of cells, including inflammation, wound healing, and microbial pathologies.^6,9,10^

While CD147 is considered an alternative target for COVID-19 infection, it also plays a role in the inflammatory response, which may contribute to the symptoms and pathogenesis of the disease.^11^ Wang et al. used the ACE2-nonexpressing cell line BHK-21 to demonstrate that SARS-CoV-2 can infect otherwise nonsusceptible tissues via endocytosis when expression of CD147 is introduced.^3^ This work provided evidence of an alternative route of viral entry and showed that CD147 can alter the virus’s tropism.^3^ Despite the weaker binding to the spike protein compared to ACE2,^12^ CD147 is a potential therapeutic target that might help prevent COVID-19 infections. However, the role of CD147 in SARS-CoV-19 as an alternative receptor of ACE2 has not been entirely confirmed.^13^ In this study, we first focused on investigating the binding of spike protein to CD147 through ELISA immunoassay and characterizing the binding force and kinetics between them by using single-molecule force spectroscopy (SMFS) -based atomic force microscopy (AFM).

To help reduce the viral entry into host cells and prevent further replication, heparin has been shown to interact with the spike protein, thereby exerting inhibitory effects and partially impairing its binding to host cell receptors.^14,15^ Heparin is a complex, naturally occurring glycosaminoglycan with anticoagulant properties, and it has been used for a long time as an antithrombotic drug, particularly for patients at high risk of thrombosis. Heparin’s anticoagulant properties can improve outcomes in these patients by reducing thrombotic complications and may offer anti-inflammatory and antiviral benefits, potentially protecting vascular endothelial cells from damage caused by SARS-CoV-2.^16^ Heparin has been observed to help inhibit the SARS-CoV-2 infection by competing with cell membrane glycosaminoglycans (GAGs) for binding to the virus’s spike protein.^15^ This competitive binding prevents the virus from attaching to host cells, thereby reducing the likelihood of infection. Additionally, heparin can hinder the cleavage of the spike protein by furin, a process essential for viral entry into cells.^15^ These findings suggest that heparin’s interaction with the spike protein disrupts multiple steps in the viral infection process, highlighting its potential as a therapeutic agent against COVID-19. Heparin is typically extracted from porcine or bovine lung mucosa. It is a heterogeneous mixture of glycosaminoglycans with slightly variable molecular weights and structures for therapeutic use in humans.^17^ The heparins can generally be classified into Low Molecular Weight Heparin (LMWH, between 4.5 kDa and 10 kDa) and Unfractionated Heparin (UFH, a mixture of low and high MW between 3 kDa to 30 kDa with an average molecular weight of approximately 15 kDa).^18,19^ Such homogeneity of molecular weight in LMWH contributes to various properties that are pharmacologically advantageous. Their anticoagulant response is much more predictable, while the half-life is longer, requiring less routine laboratory monitoring than UFH.^17^ Due to its rapid onset, short half-life, and reversibility with protamine sulfate, UFH is ideal for acute surgical settings and thromboembolic events requiring immediate and controllable anticoagulation.

Recent research has shown that both LMWH and UFH interact with the SARS-CoV-2 spike protein (SP), potentially helping to block viral entry.^15^ This interaction suggests that heparins may serve a dual role in COVID-19 treatment by providing both anticoagulant and antiviral effects.^14^ However, the precise nature of the heparin-SP interaction remains unclear.^20^ To address this, we investigated the role of heparin in modulating the interaction between CD147 and SP, aiming to better understand its effects and potential as a preventive treatment for COVID-19. Molecular simulation is a powerful tool for elucidating biomolecular interactions at the atomic level. Using molecular docking and molecular dynamics (MD) simulations, we analyzed the binding mechanisms, key interacting residues, and the dynamics of the CD147-SP complex with heparin. These insights not only enhance our understanding of SARS-CoV-2 infectivity but also contribute to identifying potential therapeutic targets to disrupt this interaction.

## Materials and Methods

### Materials

The following protein and antibodies were used: The following protein and antibodies were used: Cluster of Differentiation 147 (CD147, MW: 20.9 kDa) (Acro Biosystems, Germany, Batch number (BN): 32–217CF1–132), Full length Spike Protein, purity >98% (SP, MW: 142 kDa) (BN: BIS-BSV-COV-PR-34, Biozol, Germany), Recombinant S1 (MW: 76.9 kDa, BN: C448C1–222GF1–11Q) and S2 (MW: 60 kDa) subunits of SP, His-Tag (Acro Biosystems, Germany), anti-S1 and anti-S2 monoclonal mouse antibodies (BN:43997 and 44167 correspondingly, Gene Tex, Germany), CD147 Antibody with Alexa Flour 488 (BN: 539302, Invitrogen, Germany), Goat Anti-Mouse Antibody HRP (BN: 1025780–3, Abcam, Germany), Bovine Serum Albumin (BSA) (Roth, Germany), Unfractionated heparin (Arixtra; GlaxoSmithKline, London, UK); 3,3′,5,5′-Tetramethylbenzidine (TMB) chromogen solution (BD biosciences), Quartz crystal QX301 (5 MHz, Au-coated, AT-cut), Qsense, Biolin Scientific, Darmstadt, Germany), Cysteamine-hydrochloride (Fluka, Sigma-Aldrich, 30080, Germany), Ethanolamine (Sigma-Aldrich, E9508, Germany), Glycine (Sigma-Aldrich, 092 K0099, Germany), Glutaraldyde (Sigma-Aldrich grade I 25%), silicon nitride (Si3N4) cantilevers with a nominal spring constant k = 0.08 N/m (MikroMasch, Wetzlar Germany) and DNP-S10 (Veeco), Si3N4, k = 0.12 N/m, HS-PEG-NH2 or silane-PEG-NH2 (PEG MW 3400 Da, Nanocs, USA), amine coupling kit containing the mixture of 0.4 M 1-ethyl-3-(3-(dimethylamino)­propyl) carbodiimide hydrochloride (EDC) and 0.1 M *N*-hydroxysuccinimide (NHS) (Biocore, Uppsala, Sweden), purified CD147 (AcroBio, Heidelberg, Germany). All the chemicals were of the highest purity available and were used as purchased.

### ELISA for Confirmation of Binding

To confirm the binding of SP to CD147, CD147 (100 μL, 10 nM) was coated on a 96-well microplate and incubated overnight at 4 °C as described.^21,22^ Next, wells were washed five times with 200 μL phosphate-buffered saline (PBS) and then blocked with 200 μL of 7.5% BSA for 1 h at room temperature. 100 μL of SP, ranging from 3.9 nM to 1000 nM, was added to the wells and incubated for 1 h. Then, 100 μL of a 1:1000 dilution of the anti-S2 primary antibody was added to the wells and incubated for 1 h. After an hour, the wells were washed with PBS before incubating with goat antimouse HRP-labeled secondary antibody for 1 h. After rinsing with PBS, 100 μL of TMB substrate solution 1:1, which was prepared from TMB substrates A and B, was added to the wells. Next, the plate was incubated in the dark for 5 min. The reaction was quenched by using H2SO4. The readings were obtained for optical absorbance at 450 nm using a microplate reader and Global Gen5 software, Version 2.04, BioTek.

To investigate the effect of heparin concentration on SP-CD147 binding, 10 nM of CD147 was immobilized onto a 96-well plate as described above. Following washing and blocking steps, wells were incubated with SP/Heparin complexes that had been preformed by mixing 250 nM of SP with varying concentrations of unfractionated heparin (UFH), ranging from 3 IU/mL to 18 IU/mL, and incubating the mixture at room temperature for 1 h. Subsequent steps, including adding anti-S2 primary antibody, washing, and detection, were carried out as described above.

### Single-Molecule Force Spectroscopy (SMFS)

The triangular silicon nitride XNC12 AFM cantilevers (Mikromash) were used for this study. The square pyramidal silicon nitride cantilevers with a nominal constant of 0.08 N/m and 200 μm length were used for the SMFS experiments. Sample preparation for AFM measurements was performed as previously described.^23,24^ The cantilever and glass slides were washed with 70% ethanol and dried with air. Clean cantilevers were placed on a small piece of parafilm on a small Petri dish treated with UV-Ozone for 30 min to activate the surfaces. Then, a 50 μL droplet of 1 mg/mL silane-PEG-COOH (PEG MW 3400 Da, Nanocs, USA) was incubated on the cantilevers for 2 h at room temperature. Residual unbound material was removed by washing three times with 300 μL of PBS buffer (100 mM PBS + 100 mM NaCl at pH 7.2). After that, a 100 μL droplet of EDC/NHS (0.4M: 0.1 M dissolved in water) was added to the cantilever at room temperature for 30 min to initiate the functional group of carboxyl to facilitate the protein binding via amine bond. Then, cantilevers were washed with PBS buffer, and 70 μg/mL of SARS-CoV-2 SP was added to the cantilever for 30 min of incubation at room temperature. The residual unreacted protein was removed, and the cantilever was washed three times with 300 μL of PBS buffer, and 100 μL of 1 mg/mL ethanolamine was applied to the cantilever for 1h at room temperature to passivate the unreacted PEG-COO- groups. After washing the cantilevers with PBS buffer, the cantilevers were ready for SMFS measurements. The same protocol was used to treat bare glass substrate for the immobilization of ACE2 or CD147 receptors.

The JPK NanoWizard 3 (Berlin, Germany) was used to conduct force spectroscopy measurements on an inverted light microscope setup in contact mode. Before the experiments, the cantilever spring constants were independently determined by a thermal-tune procedure.^25^ The ACE2 or CD147 functionalized glass was placed facing up on a small Petri dish and covered with a droplet of 100 μL of PBS, pH 7.2. The laser beam was adjusted at the end of the cantilever to gain a maximal reflection signal on the position-sensitive photodetector. The functionalized cantilever with SP was approached onto the substrates functionalized with ACE2 or CD147. Force–distance (F-D) curves were recorded at a 0.2 nN set point, and >1000 F-D curves were recorded at each condition within areas of 3 μm^2^. To compare the change in the binding forces when SP interacts with ACE2 or CD147, all F-D curves were collected at a constant pulling speed of 1 μm/s to minimize variability. The use of a fixed pulling speed and consistent cantilever calibration ensured that the distribution of loading rates remained narrow across experiments. In accordance with the Bell-Evans model,^26^ unbinding forces increase with increasing pulling speed.

For the temperature dependence experiment, the glass-coated sample was submerged in PBS buffer and placed in a heating ring implemented in the AFM setup to heat samples to 37 and 40 °C. Before measurements, the samples were allowed for an additional 30 min at the given temperatures to equilibrate and minimize the thermal fluctuations. As a control, the interaction forces between SP-coated tips and PEG-coated glass were measured for the subtraction of real protein binding forces. As long PEG spacer may reduce the binding probability of ligand–receptor interaction,^27^ we optimized the bindings in SMFS by keeping AFM tips in contact with the ACE2 or CD147 coated surfaces and allowing for a rest of 0.5 to 1 s on the surfaces. The sufficient contact time facilitates the completion of SP-ACE2 or SP-CD147 complex formation. To determine the effect of heparin on the bindings, heparin was incubated on the surface coated with CD147 for 1h. After rinsing with PBS, F-D curves were recorded using SP-coated cantilevers.

To determine the effect of heparin on the binding of SP to CD147, 70 μg/mL of CD147 was immobilized on the glass substrate, whereas 70 μg/mL of SP was immobilized on a cantilever (k = 0.12 N/m) following the above protocol. The substrate was then incubated with 0.5 IU/mL UFH for 1h at room temperature before rinsing with PBS to wash off the unbound UFH. Finally, the SP-coated cantilever was brought to the substrate for interaction with CD147/UFH in PBS at room temperature.

### Data Analysis of Forces

The JPK data processing software (version 4.4.18+) was used to process the generated force curves and generate different adhesion force values for every point taken. The JMP 17 statistical analysis software was used for ANOVA and Tukey-Kramer all means comparison test to evaluate the significance (p-values at α = 0.05) of the data collected and generate adhesion force distribution curves and other plots. We analyzed only F-D curves that displayed polymer stretching behavior characteristic of Worm-Like Chain (WLC) or Freely Jointed Chain (FJC) models. F-D curves that did not show such features or that exhibited forces below 30 pN were excluded, as these signals are considered to fall below the detection limit of the AFM and may reflect nonspecific or background interactions. This selection ensures that the analyzed rupture events reflect meaningful molecular interactions within the expected force and distance range.

### Binding of Heparin to S1/S2 Subunits Determined by QCM

To understand if S1 or S2 subunits bind to heparin or CD147, the mass-sensitive detection method, Quartz Crystal Microbalance (QCM), was used. Sample preparation for QCM experiments was performed as previously described.^21^ Briefly, the Au-coated quartz crystal QX301 chips (5 MHz, Au-coated, AT-cut, Qsense, Biolin Scientific, Darmstadt, Germany) were first treated with UV/ozone for 10 min to remove surface contaminants, followed by immersion in a 5:1:1 solution of H_2_O, NH_3_, and H_2_O_2_ in an ultrasonic bath for an additional 10 min to ensure thorough cleaning. After rinsing with water and drying with a nitrogen flow, the chips were mounted in a four-chamber module, with each chamber mounted with a single chip and connected to the pump (High Precision Multichannel Dispenser, ISMATEC). The gold chips were incubated with 2 mL of 30 mM cysteamine hydrochloride for 1h at room temperature to form self-assembled monolayers. After incubation, the chips were washed thoroughly with deionized water. The chips were then equilibrated by washing them with PBS for 5 min. The next step involved the activation of the gold surface by the addition of 2 mL glutaraldehyde solution (30 mM) to each chamber and incubation for 1h, followed by an additional 5 min PBS wash. After that, 500 μL of solution with 125 nM S1 or S2 protein or 50 nM CD147 was incubated for 20 min on each chip. To avoid nonspecific bindings, a PBS wash for 5 min before adding 2 mL ethanolamine (10 mM) for 1h incubation was added to block free aldehyde (−CHO) groups. After PBS wash for 5 min, unfractionated heparin (UFH) of different concentrations (3 or 9 IU/mL) was added and incubated for 20 min, followed by a final 5 min PBS wash. The real-time resonant frequency change was recorded at the third overtone due to stability constraints at a higher order. QCM real-time resonant frequency changes were observed on Qsoft software (version 2.5.22.707, Q sense, Biolin Scientific, Europe) and analyzed using the Sauerbrey equation (which has a standard value of 17.7 ng/cm for the 5 MHz crystal)^28^ through Qtools software as described.^22,29^ All preparations were performed at room temperature, whereas QCM measurements were performed at 25 °C under continuous flow at a pumping speed of 100 μL/min. Data was further analyzed using Excel and Origin 7.5.

### Molecular Simulation Method

Initial protein structures for the S1 subunit of the SARS-CoV-2 spike protein (S1), human CD147 receptor, and heparin were obtained from the Protein Data Bank (PDB), which is a repository of experimentally determined protein structures.^30^ By extracting their respective crystal structures with accession codes 6VSB for the S1 and 6LZ0 for the human CD147 receptor,^31,32^ we ensured that these structures were based on high-quality experimental data. For simulations, we selected a heparin molecule with a chain length of dp24, i.e., a specific heparin fragment with a degree of polymerization (dp) of 24, meaning that the fragment is composed of 24 sugar units (saccharides). This specific heparin structure was obtained from the PDB (PDB: 3IRJ),^33^ which represents its experimentally determined conformation. Protein–protein docking is a crucial step in understanding the molecular interactions between S1 and CD147. We employed the HDock server, an efficient and accurate docking tool.^34^ Upon completion of docking simulations, we analyzed the resulting ensemble of predicted S1-CD147 complexes. To narrow down the most likely complexes and minimize computational resources for subsequent molecular dynamics (MD) simulations, we selected only the top 3 most favorable complexes based on their binding affinity scores provided by the HDock server. These three complexes were considered potential candidates for representing the S1-CD147 interaction, and further MD simulations were performed to investigate the stability and conformational changes of the complexes over time.

The CD147-dp24 interactions were not subjected to protein–protein docking using HDdock due to the flexibility of heparin and its ability to adopt multiple conformations in solution, which makes it challenging for traditional docking methods to accurately predict binding modes. Instead, we generated several initial geometries for the CD147-dp24 complex to account for the potential conformational flexibility of both proteins and their interactions. This process involved randomly placing dp24 at several propositions close to the CD147. Each configuration underwent a geometry optimization process to obtain stable conformations. The most stable conformation among these was then selected for further simulations. This approach allowed us to capture a more comprehensive representation of the binding process between heparin dp24 and the CD147 receptor.

To examine the impact of heparin on the interaction between the S1 and CD147, ternary complexes were constructed starting from the most stable S1-CD147 complex as a reference structure. The heparin dp24 molecule was randomly placed around this reference complex while maintaining an appropriate distance between the spike protein and heparin to mimic their possible interaction sites. Following random placement, the geometries of the resulting ternary complexes were optimized using molecular mechanics minimization techniques, which aimed to alleviate any steric clashes or unfavorable interactions that might have been introduced during the random placement process. The most stable geometries obtained after optimization were used as starting poses for the MD simulations and binding free energy calculations.

MD simulations provide insights into conformational changes and dynamic properties of proteins, as well as their interactions with other molecules. To perform MD simulations for the S1-CD147 complex and heparin-CD147 complex, we utilized Schrödinger’s Desmond software, which employs the OPLS force field^30,35^ to model interactions of both proteins and ligands. The structure obtained from docking was preprocessed to assign bond orders and add hydrogens. Then, it was solvated in a box with a buffer distance of 10 Å to the boundary using the TIP3P water model. Long-range electrostatic interactions were efficiently treated using these molecular dynamics simulations, which utilize the Smooth Particle Mesh Ewald (SPME) algorithm to accurately calculate short-range nonbonded interactions with a 9Åcutoff. The time step of 2 fs was employed. The M-SHAKE algorithm was used to constrain all hydrogen-bonded interactions. Following the default relaxation protocol in Desmond,^36^ we equilibrated the systems to ensure that they reached a stable state before initiating production runs. This process involved energy minimization, heating, and equilibration steps to eliminate steric clashes and enable the systems to adapt to the simulation conditions. To mimic the human body’s environment, we added a 0.15 M NaCl salt solution to neutralize the system and maintain electrostatic balance. The temperature was set to 310 K to represent the human body’s physiological temperature. Three independent 200 ns production runs were performed for each complex (Figure S4). For the S1-CD147 complex, the initial structure was obtained from the docking studies. In contrast, for the heparin-CD147 complex, we generated an initial structure randomly to investigate the binding behavior of heparin with CD147 without any prior knowledge of the interaction. The number of atoms ranged from approximately 350,000 to 400,000 for the S1-CD147 complexes and around 100,000 for the dp24-CD147 systems. Figure S5 depicts the protein–protein interaction analysis between the spike protein (S1) and CD147 during the last nanosecond of the MD simulation.

The Molecular mechanics with generalized Born and surface area solvation (MM/GBSA) method^37^ within Amber Tools^38^ was employed to calculate the binding energy. In the MM/GBSA method, the binding free energy between S1 and CD147 is calculated as (eq 1–2):

1 $$\Delta G_{bind}=G_{complex}-\left(G_{S1}+G_{CD147}\right)$$

2 $$G=E_{Internal}+E_{vdW}+E_{Elec}+G_{Polar}+G_{non‐Polar}$$

In calculating the total energy (G) of a system, van der Waals (EvdW) and electrostatic (EElec) energies are determined in vacuum conditions (eq 3). The polar (GPolar) and nonpolar (Gnon-Polar) terms, on the other hand, account for the free energy changes associated with solvation- the process of transferring a system from a vacuum to a specific solvent environment. The GPolar term represents the reduction in electrostatic energy that occurs when charges are transferred from a vacuum into a solution. Numerically, GPolar is obtained using the Generalized Born (GB) approximation, which estimates the electrostatic interaction between solute and solvent by simulating how charges are screened in a medium with a given dielectric constant. Typically, the solute dielectric is set low to mimic a low-polarity interior, while the solvent dielectric is high (e.g., 80 for water) to reflect strong polarization.

In solvents, the electric field experienced by these charges is significantly reduced, on average, by a factor of 8–10 compared to vacuum conditions. Consequently, the GPolar and EElec terms nearly cancel each other in the total energy calculation, reflecting the stabilizing effects of solvation on charged species within the system.

The calculation of Gnon-Polar is based on the solvent-accessible surface area (SASA) model, reflecting the hydrophobic effect and van der Waals interactions with the solvent. It is modeled using an empirical linear relationship: Gnon-Polar = γ·SASA + β, where γ (0.0072 kcal/mol/Å2) and β (0.0 kcal/mol) are transfer-free energy coefficients derived from experimental data on the solvation of nonpolar molecules.^37^ These coefficients effectively encode the energetic cost of forming a cavity in the solvent and the nonelectrostatic stabilization provided by solvent–solute contact. This model estimates the free energy change associated with solvation as the product of the solute’s surface area exposed to the solvent and a surface tension factor that accounts for the dispersion forces between solute and solvent molecules.

3 $$\Delta G_{bind}=\Delta E_{vdW}+\Delta G_{Elec,Polar}+\Delta G_{non‐Polar}$$

During the MD simulations, a total of 100 representative frames were extracted from the final nanosecond of each trajectory to ensure that the selected conformations captured the most relevant and stable interactions between the biomolecules. These frames were then subjected to calculations using the MM-GBSA method, which allowed for the estimation of binding free energy as an average value for each complex.

Steered Molecular Dynamics (SMD) simulations were performed using GROMACS (version 2024.2) to investigate the unbinding process between the SARS-CoV-2 S1 protein and the CD147 receptor. The total system consisted of approximately 900,000 atoms. The LINCS algorithm^39^ was used to constrain bond lengths, allowing for a 2 fs integration time step. The SMD simulations were conducted by applying a harmonic potential to the center of mass (COM) of the SARS-CoV-2 S1 protein, with a pulling rate of 5 nm/ns along the vector connecting the COMs of the S1 protein and the CD147 receptor. The force constant for the harmonic potential was set to 500 kJ/mol/nm2. Each pulling simulation was performed for 3 ns and repeated 50 times to ensure statistical robustness.

During each simulation, the force profile and rupture force were recorded. The rupture force was defined as the maximum force observed during the unbinding event. To examine the impact of heparin on the interaction between the SARS-CoV-2 spike protein subunit S1 and CD147, two distinct sets of simulations were conducted: one in the absence of heparin and another in its presence. By comparing the average rupture forces obtained under these two conditions, we aimed to assess the effect of heparin on the binding affinity between S1 and CD147.

## Results

To elucidate the binding characteristics of SP to CD147, a combination of experimental and computational methods was employed. ELISA quantified the binding affinity, while single-molecule force spectroscopy (SMFS) provided qualitative insights into the binding forces of the SP-CD147 interaction. SMFS further revealed binding kinetics, including thermal off-rates and the effects of temperature on binding strength. The influence of heparin on SP-CD147 binding was assessed using ELISA, SMFS, and QCM. Additionally, molecular simulations were conducted at the atomic and single-molecule levels to gain deeper insights into the interaction dynamics.

### ELISA Quantification of SP-CD147 Binding

To confirm the binding of the full-length spike protein (SP) to CD147, we performed a sandwich ELISA. CD147 (10 nM) was immobilized on a 96-well ELISA to capture SP at concentrations up to 1000 nM. A secondary anti-S2 antibody conjugated with an enzyme as the detection molecule, as using an anti-S1 antibody did not provide a good detection signal (Figure S1). By adding TMB substrate, the chromogenic reaction converted the enzyme into a colored product, which could be measured as the optical density (OD) at 450 nm absorbance using a plate reader. Different controls were carried out to confirm the binding. The results showed an increased OD as SP concentration increased (Figure 1).

**Figure 1:**
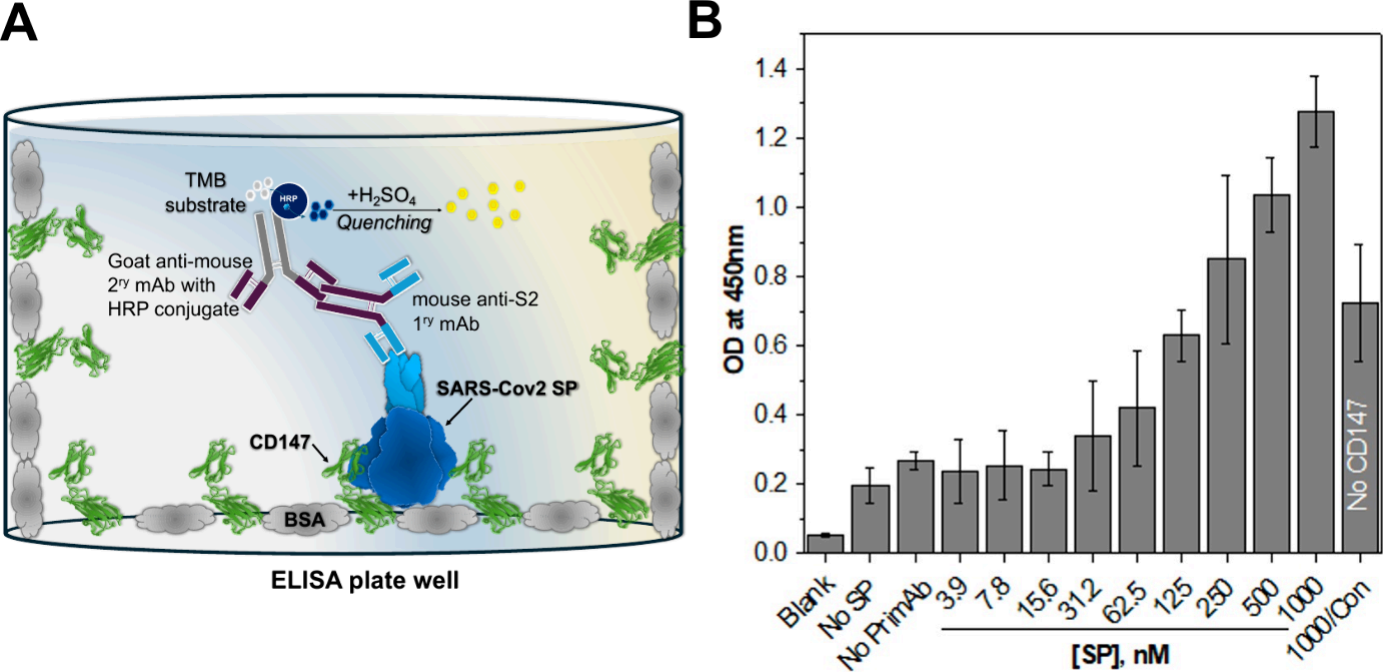
Binding of SP to CD147 in ELISA. (A) The cartoon illustrates the ELISA setup for detecting SP-CD147 binding. (B) Titration of SP from 3.9 to 1000 nM into wells coated with 10 nM CD147 shows an increase in binding as SP concentration increases, and the signal is higher than that of the control (1000/Con = absence of CD147, tested at 1000 nM SP). Error bars represent the mean standard deviation of three experiments (*n* = 3 repetitions).

The absorbance values increased from 0.24 ± 0.08 at 3.9 nM from the lowest concentration of SP to 1.3 ± 0.08 at the highest concentration of 1000 nM. The results confirm the binding of SP to CD147. The anti-S2 instead of the anti-S1 antibody was selected for detection, as upon binding, the CD147 may block other available epitopes on S1 for anti-S1 antibodies. As a result, weak OD could be observed if the anti-S1 antibody is used. In contrast, the S2 subunit is involved in the fusion of the viral and host membranes and is less directly involved in receptor binding, allowing full access to the anti-S2 antibody. The blocking control value demonstrates BSA’s ability to minimize nonspecific binding, thus providing a clearer distinction between nonspecific interactions (in the blocking control) and specific binding between CD147 and SP in the assay tested at 1000 nM SP. Despite blocking with 7.5% BSA, a moderate background signal was observed in the control condition without CD147, likely due to nonspecific binding of spike protein to the ELISA plate or residual impurities. However, the optical density in the presence of CD147 was significantly higher, confirming specific SP-CD147 interaction. Based on these results, 10 nM CD147 and 250 nM SP as optimal concentrations were selected for further ELISA tests, as they showed reasonable OD signals for detection.

### Binding Forces between SP and CD147

The interaction force between the full-length SP and ACE2 or CD147 was measured through SMFS-based AFM. The CD147 was immobilized on a glass slide and probed with an SP-coated nitride oxide cantilever to measure adhesion forces upon retraction (Figures 2A-B). Various cantilevers and surface glass controls (Figure S2) were used to determine an average value of nonspecific adhesion force (30 pN), which served as a criterion to distinguish specific adhesion forces in the statistical analysis of the SMFS experiments. The SP-CD147 interactions reported force values ranging from 32 to 78 pN, but the estimated average force was 41.9 ± 0.7 pN (Figure 2C). The ACE2 showed comparable interaction force to the spike protein than CD147, as the ACE2 interaction forces ranged between 35 and 72 pN and averaged around 42.7 ± 0.9 pN. Although both demonstrated significantly higher adhesion forces than the PEGylated glass negative control (17.2 ± 0.4 pN, p-Value = < 0.001; α = 0.05), both receptors showed no significant difference in the mean adhesion force when compared (p-Value = 0.9279; α = 0.05).

**Figure 2:**
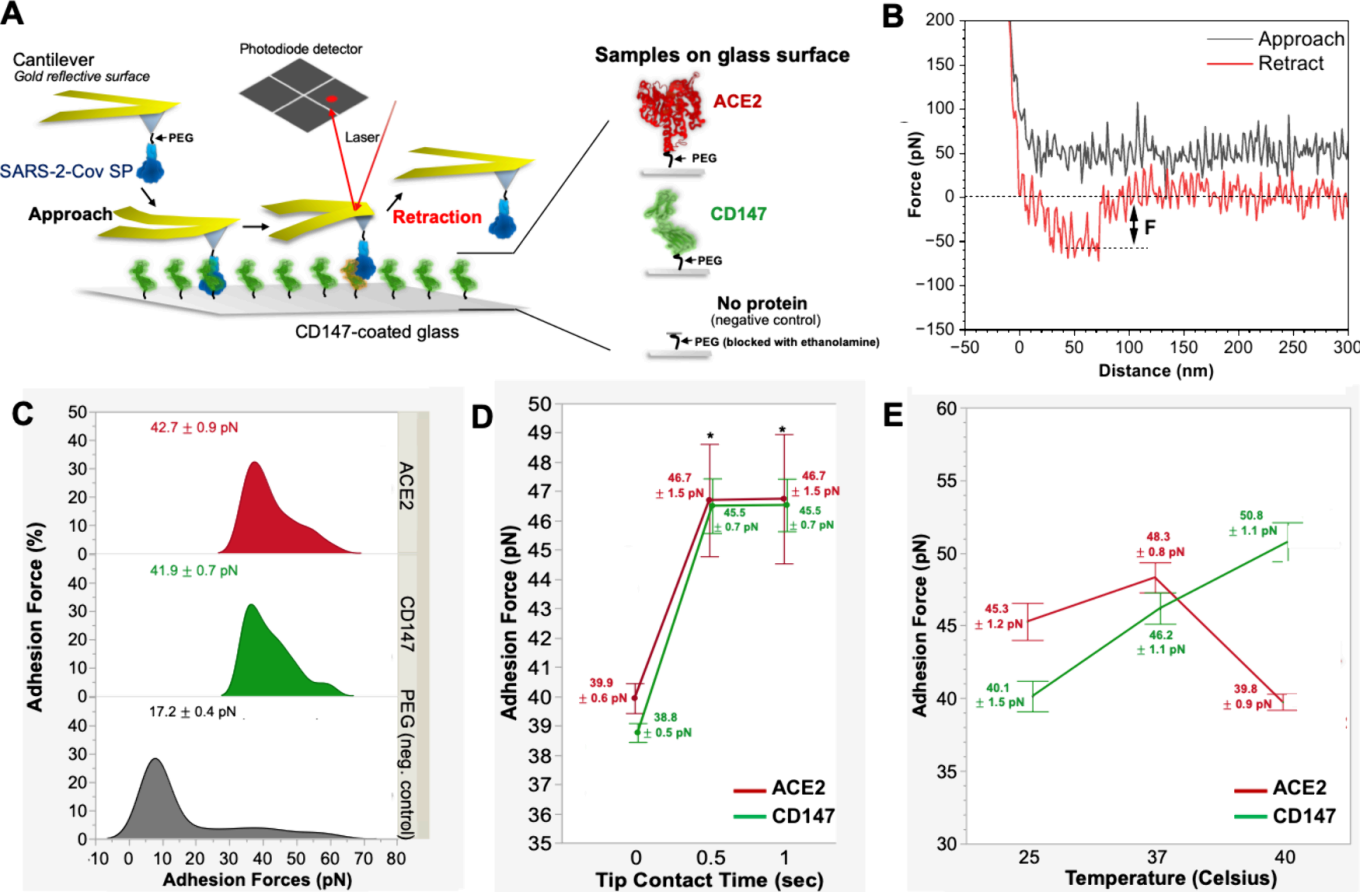
Measuring the binding force between SP and CD147 using SMFS. (A) SMFS setup: through a PEG linker, the SARS-CoV-2 SP is immobilized on the AFM cantilever’s probe while CD147 or ACE2 proteins are immobilized on the glass. The cantilever-SP was approached on the glass surface displaying ACE2, CD147, or just PEG, in which −COOH groups were blocked with ethanolamine (= negative control). (B) A typical F-D curve illustrates the interaction between SP and CD147 as the cantilever approaches the substrate (blue curve), and the binding force (F) is measured by retracting the cantilever-SP from the sample (red curve). (C) Histogram distributions of binding forces collected from all F-D curves between SP and CD147 (green) or ACE2 (red) are higher than those measured on PEGylated glass (gray), while Gaussian fits on these data allowed the determination of mean adhesion force ± standard errors. (D) Mean adhesion forces at different tip contact times were measured with (red) ACE2 and (green) CD147 with standard error whiskers (±standard errors) determined with a ONEWAY ANOVA statistical analysis: (E) Effect of temperature on the binding between SP and ACE2 (red) or CD147 (green) based on mean adhesion force as a function of temperature: *n* = 3 repetitions.

However, the results showed no significant difference between ACE2 and CD147 at the tip/surface contact time of zero seconds. Here, the time of tip–surface contact may not be sufficient for a complete binding interaction between SP and targets. In SMFS experiments, the tip–sample contact time is a critical parameter influencing the measured interaction forces. During its contact period, molecular bonds form between functionalized AFM tips and target molecules on the sample surface. However, intensively prolonged contact times can allow for establishing multiple interactions that affect the analysis of a single molecular interaction. In contrast, shorter contact times may limit bond formation to weaker or transient interactions. Understanding the effect of tip contact time on the sample surface is essential for accurately interpreting binding force profiles and the dynamics of molecular interactions in SMFS experiments. As longer contact time causes multiple bindings, the tip contact time here was investigated in a range between 0 to 1 s (s). When the tip contact time is 0s, both forces induced by binding of SP to ACE2 and CD147 showed comparable to the previous experiment with average adhesion force values in both ACE2 (39.9 ± 0.6 pN) and CD147 (38.8 ± 0.5 pN) (Figure 2D). In contrast, when tip contact time was increased to 0.5s and 1s, both forces showed significantly higher mean values (p-Value <0.001; α = 0.05) and the force saturated from 0.5s. However, no significant difference between the interaction with ACE2 and CD147 at 0.5s and 1s dwelling levels (p-value 0.997; α = 0.05). The low adhesion forces observed at tip contact times shorter than 0.5 s are likely due to insufficient time for the full formation of interactions between the spike protein and its receptors, ACE2 or CD147.

Temperature plays a crucial role in modulating the binding interactions between biomolecules, influencing both kinetic and thermodynamic properties. In the context of SP binding to CD147, SMFS enables detailed characterization of how temperature affects binding strength and stability. In principle, elevated temperatures can weaken interactions by accelerating bond dissociation and increasing molecular flexibility, while lower temperatures may enhance binding stability by reducing thermal fluctuations. Investigation of the temperature dependence of SP-CD147 interactions provides valuable insights into their binding dynamics and stability under physiological and pathological conditions. Here, the effect of temperature ranging from 25 to 40 °C (fever condition) on adhesion forces between the SP and ACE2 and CD147 was evaluated. The SP binding ACE2 showed consistent adhesion forces at different temperatures, ranging from 35 to 80 pN. When the mean adhesion forces were compared in a ONEWAY ANOVA Tukey-Krammer all-means comparison analysis, no significant differences between 25 and 37 °C were found (p-value = 0.1084; α = 0.05). However, the mean adhesion force for both 25 °C (45.3 ± 1.2 pN) and 37 °C (48.3 ± 0.8 pN) was significantly higher than at 40 °C (39.8 ± 0.9 pN; p-value = 0.0011 for 25 °C; p-value <0.0001 for 37 °C; α = 0.05). The results confirm that the ACE2 target has consistent interaction with SP at temperatures up to 37 °C. Our findings agree with previous studies as SP decreases interaction with ACE2 receptors in fever temperature conditions.^40^

On the contrary, CD147 analysis shows more adhesion forces with increasing temperatures (p-values = 0.0134 for 37 °C vs 40 °C, = 0.0038 for 37 °C vs 25 °C, and < α; α = 0.05). The mean adhesion force comparison analysis finds significantly higher adhesion forces between SP and CD147 with each increasing temperature (Figure 2E). ACE2 shows stronger adhesion than CD147 at 25 °C and 37 °C, but its force drops significantly at 40 °C, falling below that of CD147. This suggests that while ACE2 interaction weakens with rising temperature, CD147 becomes more adhesive above 37 °C. Even though CD147’s mean adhesion force (46.2 ± 1.1 pN) at 37 °C was slightly lower than ACE2 (48.3 ± 0.8 pN), the Tukey-Kramer all means comparison analysis showed they are not significantly different from each other (p-Value = 0.1840; α = 0.05). This suggests that increasing temperatures may play a role in enhancing the binding interface of CD147 with SP while decreasing the binding strength of SP with ACE2. At 40 °C, there is an overall greater population of higher adhesion force events for CD147 (50.8 ± 1.1 pN) as compared to ACE2 (39.8 ± 0.9 pN). While ACE2 presents more adhesion forces at 25 and 37 °C, the CD147 ONEWAY ANOVA Tukey-Kramer all means comparison analysis shows significantly higher than ACE2 at 40 °C (p-value <0.0001; α = 0.05). Interestingly, the adhesion force pattern is inverse at higher temperatures among biological targets, which may suggest the possibility of interacting with alternative receptors based on environmental conditions.

### Effect of Heparin on the Binding of Spike Protein to CD147

#### QCM Determination of Binding of Heparin to S1/S2 Subunits and CD147

To identify if heparin blocks the binding of SP to CD147, unfractionated heparin (UFH) was first tested with SP or CD147 alone to determine possible interactions. To investigate this, QCM, a real-time detection technique that reports binding interactions over time through changes in frequency, was employed. The quartz crystal (gold chip) was immobilized with 125 nM of S1 or S2 before adding 3 IU/mL UFH for binding. The bound UFH resulted in mass changes that are sensitively detected at a resolution range of ng/cm^2^. In this method, a larger QCM frequency change results from a greater mass change, which translates into stronger interaction. Figure 3 shows the change in frequency over a real-time course as the added UFH interacts with the S1 subunit coated on the sensor surface. The frequency shift varied depending on the chemical reagent added (Figure 3A). Enlargement curves illustrate the interaction between the S1 subunit and heparin. Representative QCM spectra showed distinct frequency shifts upon addition of unfractionated heparin (UFH) to sensors coated with either the S1 (blue) or S2 (light blue) subunits, indicating stronger binding of UFH to S2 than to S1 (Figure 3B). Quantification of mass changes from two independent experiments revealed increased binding of both S1 and S2 to 9 IU/mL UFH compared to 3 IU/mL (Figure 3C). Notably, S1 showed strong binding to CD147, which was further enhanced in the presence of UFH, suggesting a mediating role of UFH in this interaction (Figure 3C).

**Figure 3:**
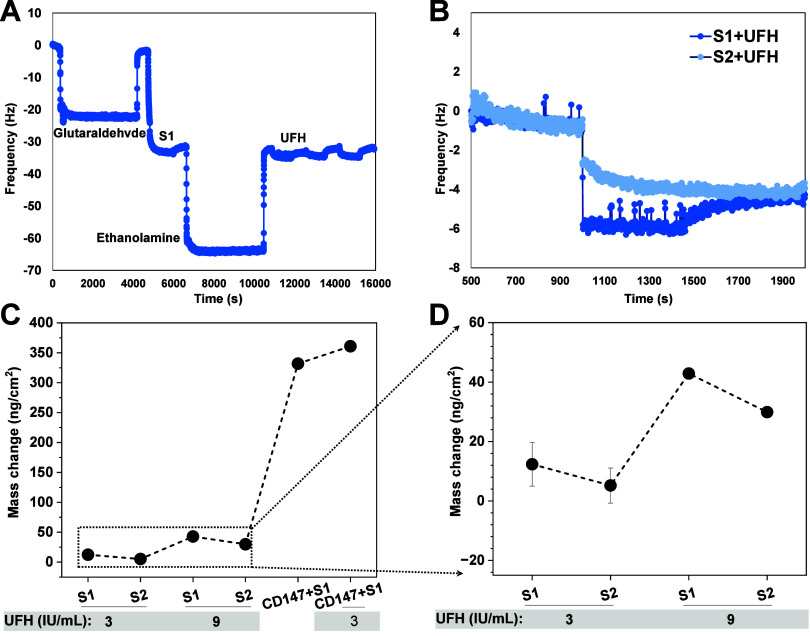
Interaction between S1 subunit and unfractionated heparin (UFH) in QCM. (A) Real-time QCM spectra of the interaction between S1 and UFH. (B) Typical QCM spectra show frequency shift differences when heparin was added to an S1 (blue) or S2 (light blue)-coated sensor. (C) Quantification of mass added due to heparin binding to S1, S2, and CD147. (D) Enlargement shows higher bound mass caused by S1 at both 3 and 9 IU/mL UFH. *n* = 2 independent experiments.

In this experiment, a change in frequency was observed after the S1 or S2 protein had already been immobilized on the gold chip, followed by the addition of 3 IU/mL heparin. Subsequent washes using PBS demonstrated that the binding was both stable and specific due to the retention of an almost similar signal to postwash. These results show that heparin binds differently to the S1 and S2 subunits of the SP of SARS-CoV-2. The larger mass change from the S1 + Heparin interaction (12.4 ± 5.1 ng/cm^2^) indicates that heparin binds more firmly to the S1 than to the S2 (7.3 ± 5.0 ng/cm^2^), thus suggesting a stronger affinity. The sudden jump of S1 when binding to Heparin, in contrast to S2, shows an exponential mass increase over time, indicating a higher affinity of S1 to heparin than S2. These findings agree with the previous study, underlining the complex interaction of heparin and SP, showing its ability to bind different regions of the viral protein.^41^

To further understand the role of heparin, different UFH concentrations up to 18 IU/mL were coincubated with 250 nM SP before being added to CD147-coated 96-well plates. The results showed OD increase at concentrations ≥ 12 IU/mL (arrow, Figure S3), however, it lacked of significant difference when compared with the case in the absence of heparin (P = 0.808). Similarly, the frequency shift observed for CD147 upon heparin addition was also significantly larger, indicating a far larger mass change upon binding. As ratio proportionality relates the frequency and mass, the larger frequency shift indicates a greater extent of interaction. The frequency changes indicate that something is binding to the surface of the gold, as supported by the mass difference bar chart in Figure 3C.

#### SMFS Determination of Binding between SP and CD147 in the Presence of Heparin

To understand the effect of heparin on the spike protein binding to CD147, their interaction force in the presence of heparin was further determined by SMFS (Figure 4). CD147 was immobilized on the substrate, and then the surface was incubated with 0.5 IU/mL UFH. After rinsing to remove unbound UFH, the SP coated on the AFM cantilever was brought in contact with CD147/Heparin complexes on the substrate for interaction. The system in the absence of heparin was also performed for comparison. The results showed that SP interacted with CD147/Heparin complexes with higher binding forces (Figure 4A, right) compared with CD147 alone (Figure 4, left). The variation of the force distribution also increased after the involvement of the heparin. The addition of heparin to CD147 resulted in a higher rupture force, indicating that heparin bound to CD147 even after washing. The increase in force between SP and CD147/Heparin complexes suggests that heparin acted as a mediator between spike protein and CD147, enhancing the binding force among these two proteins.

**Figure 4:**
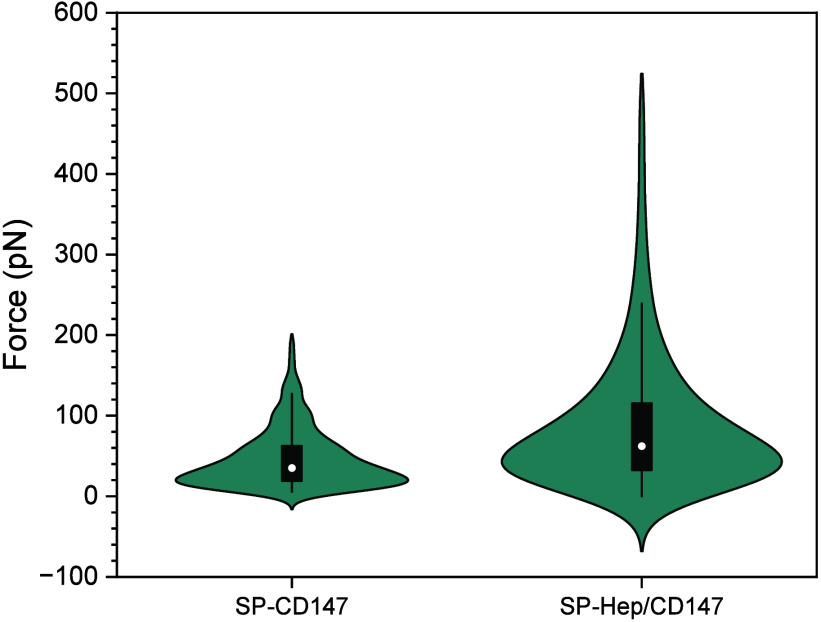
Effect of heparin on the binding forces of SP to CD147 determined by SMFS. Heparin-bound CD147 caused an increase in the binding force of spike protein to CD147 (right) compared to no heparin added (left). The central black bar represents the interquartile range with the median marked by a line, while the white dot indicates the mean of the data. All measured forces are positive while the apparent negative regions in the violin plots arise solely from the density estimation process.

### Molecular Modeling and Simulations

Figure 5 presents snapshots illustrating the binding interactions between S1, CD147, and heparin, derived from 200 ns of MD simulations. To ensure the reliability of our findings, three independent simulations were performed for each complex. The stable nature of these complexes is evident, as no separation or disassembly was observed throughout the MD simulations, indicating a robust binding interaction between them. Figure 5 (A-C) shows snapshots of the S1–CD147 complex, each derived from a different initial docking pose generated by HDOCK and subsequently refined through molecular dynamics simulations. This representative frame illustrates the proximity and interactions between the S1 domain and CD147 receptor, highlighting the critical role of this interaction in the virus’s entry into host cells. The figure demonstrates three distinct positions of CD147 binding to the S1 domain of the Spike protein.

**Figure 5:**
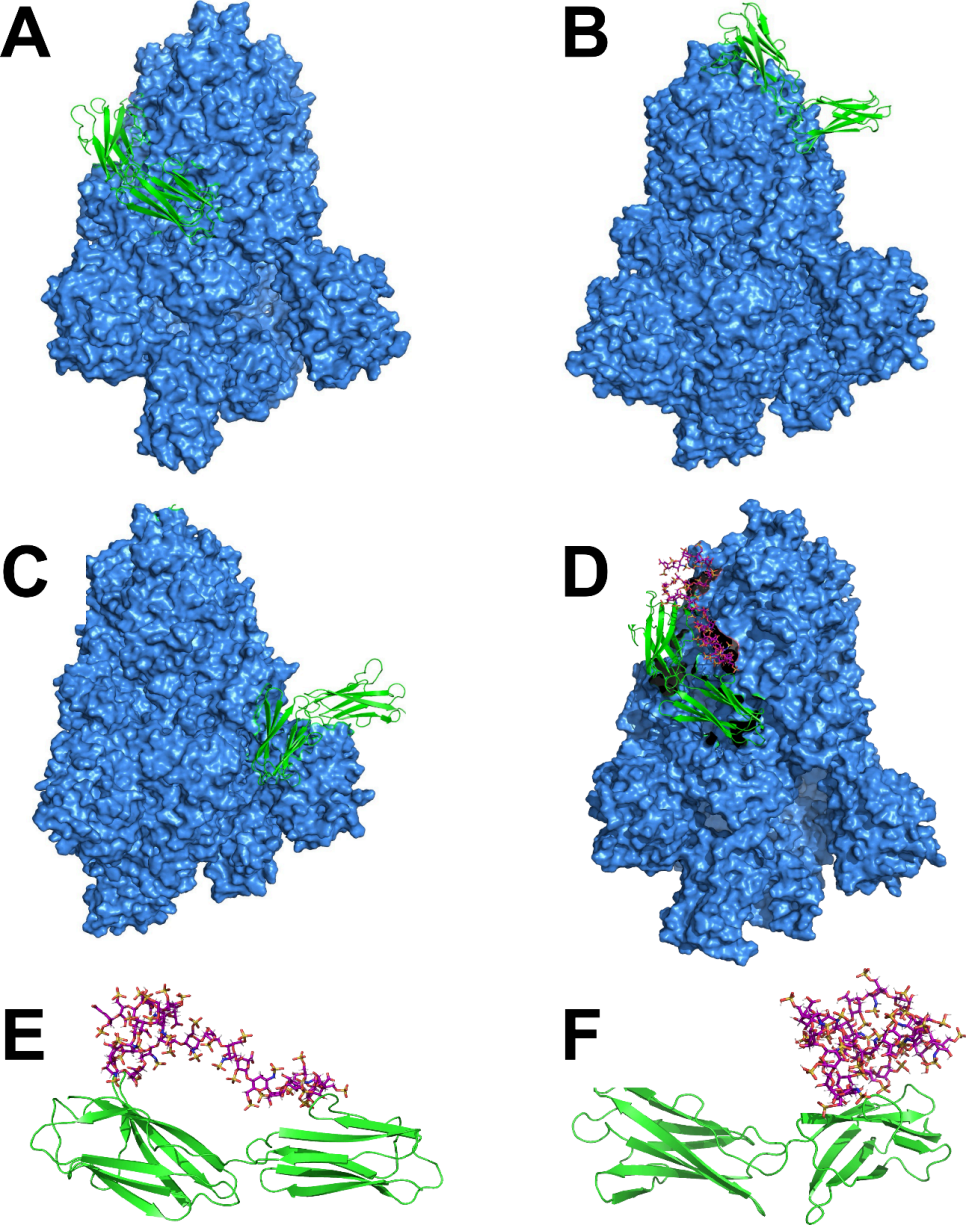
Snapshot of SARS-CoV-2 Spike protein (S1 domain)-CD147 complex obtained after 200 ns simulations from MD simulations. (A-C) displays representative frame highlighting the proximity and interactions between the S1 domain and the CD147 receptor, (D), and with dp24 heparin included and (E-F) shows the interactions between the CD147 receptor and heparin molecule. Water solvent and other ions have been removed for clarity.

To assess the stability of the S1-CD147 complex during molecular dynamics simulations, the root-mean-square deviation (RMSD) of the complex was analyzed. For instance, Figure S4 (Supporting Information) displays the time evolution of RMSD for the S1-CD147-A complex for three independent runs, illustrating how this measure of structural fluctuation changes throughout the simulation. The stable RMSD profile, with values remaining below 6 Å in the simulation, indicates that the S1 domain maintains a consistent binding interaction with the CD147 receptor. This observation suggests that the association between the S1 domain and the CD147 receptor is robust and stable under the conditions of the MD simulations. Particularly, Figure 5D reveals the binding positions of the three components: S1, CD147, and heparin. It is important to note that there are close contacts between all three compounds. The structural analysis suggests that heparin prefers to bind at the interface between the S1-CD147 complex, effectively maximizing the van der Waals interactions among the three components, which contributes to the enhanced stability of the overall binding complex.

Figures 5E and 5F present snapshots depicting two distinct conformations of the dp24-CD147 complex, highlighting varying levels of interaction between the two components. In one conformation (Figure 5E), the interaction between dp24 and CD147 is weak, while in the other (Figure 5F), the interaction is stronger. Both conformations remain stable throughout the 200 ns of MD simulations, indicating that the dp24 molecule exhibits remarkable flexibility. This adaptability allows dp24 to adjust its geometry according to the molecular partner it interacts within the solution.

Table 1 summarizes binding energy analysis for different systems obtained using the MM-GBSA method. For the S1-CD147 complexes (A, B, and C), the vdW interactions substantially contribute to the binding energy, indicating that these forces play a significant role in stabilizing the complexes. The electrostatic, polar, and nonpolar contributions are relatively small. This indicates that the vdW interaction is most important for S1-CD147 complexes. Across all three S1-CD147 systems, the overall binding free energies (Δ*G*_*bind*_) are negative and relatively large (ranging from −108 to −133 kcal/mol), suggesting that the interactions between S1 and CD147 are energetically favorable. In comparison, the dp24-CD147 complex has a lower Δ*G*_*bind*_ of – 37 kcal/mol. This lower binding energy likely reflects the smaller size of the dp24 and the reduced number of atoms in the complex, which results in fewer intermolecular interactions. Therefore, while the interaction is still favorable, the total binding energy is expected to be lower due to the more limited interaction interface.

**Table 1:** Binding Energy Analysis for Different Systems Obtained from the MM-GBSA Method^a^

**System**	**ΔE_vdW_**	**ΔG_Elec,Polar_**	**ΔG_non‑Polar_**	**ΔG_bind_**
S1-CD147.A	–142	28	–19	–133
S1-CD147.B	–117	23	–14	–108
S1-CD147.C	–113	19	–16	–110
S1-dp24	–39	10	–6	–36
dp24-CD147.A	–37	6	–6	–37
dp24-CD147.B	–51	11	–8	–47
(S1.dp24)-CD147	–170	42	–24	–152
(S1.CD147)-dp24	–77	21	–12	–67

a All values are in kcal/mol. Systems include: S1-Spike protein (SARS-CoV-2), CD147-receptor, and Heparin (dp24) - chain length of 24.

In the presence of heparin, the binding free energy between S1 and CD147 is significantly enhanced, reaching – 152 kcal/mol in the (S1.dp24)–CD147 complex. This value is notably more favorable than the most stable binary S1–CD147 interaction observed in isolation (−133 kcal/mol), suggesting that dp24 may facilitate or stabilize the S1–CD147 association. The flexibility and charge distribution of dp24 likely contributes to this enhanced binding, possibly by promoting additional contacts or conformational alignment between the partners.

When examining the interaction of dp24 with CD147 alone, the binding free energies are considerably lower, consistent with fewer interaction sites. The dp24–CD147.A complex exhibits a binding energy of – 37 kcal/mol, while the dp24–CD147.B system shows a moderately stronger interaction at – 47 kcal/mol. Notably, in the (S1.CD147)–dp24 configuration, dp24 displays a stronger binding energy of – 67 kcal/mol. This increase likely arises from dp24 interacting with both S1 and CD147 simultaneously within the ternary complex, enhancing its binding affinity relative to binary systems. These results suggest that while dp24 alone binds relatively weakly, it can act cooperatively to stabilize multiprotein interactions in the full complex.

Figure 6A depicts an illustrative example of the force profiles obtained from SMD simulations conducted on S1-CD147 complexes, both with and without the presence of heparin. The significantly elevated pulling rates employed in these simulations result in higher forces being observed as compared to those measured in AFM experiments. Nonetheless, the relative magnitudes of the forces provide valuable information for comparing the mechanical properties of the S1-CD147 complex under two different conditions. As shown in Figure 6B, the presence of heparin increases the rupture force, suggesting that it is exerting mechanical stability over the S1-CD147 complex. The presence of heparin during the unbinding process significantly elevates the average rupture force, resulting in an approximate increase of around 400 pN compared to the scenario without heparin.

**Figure 6:**
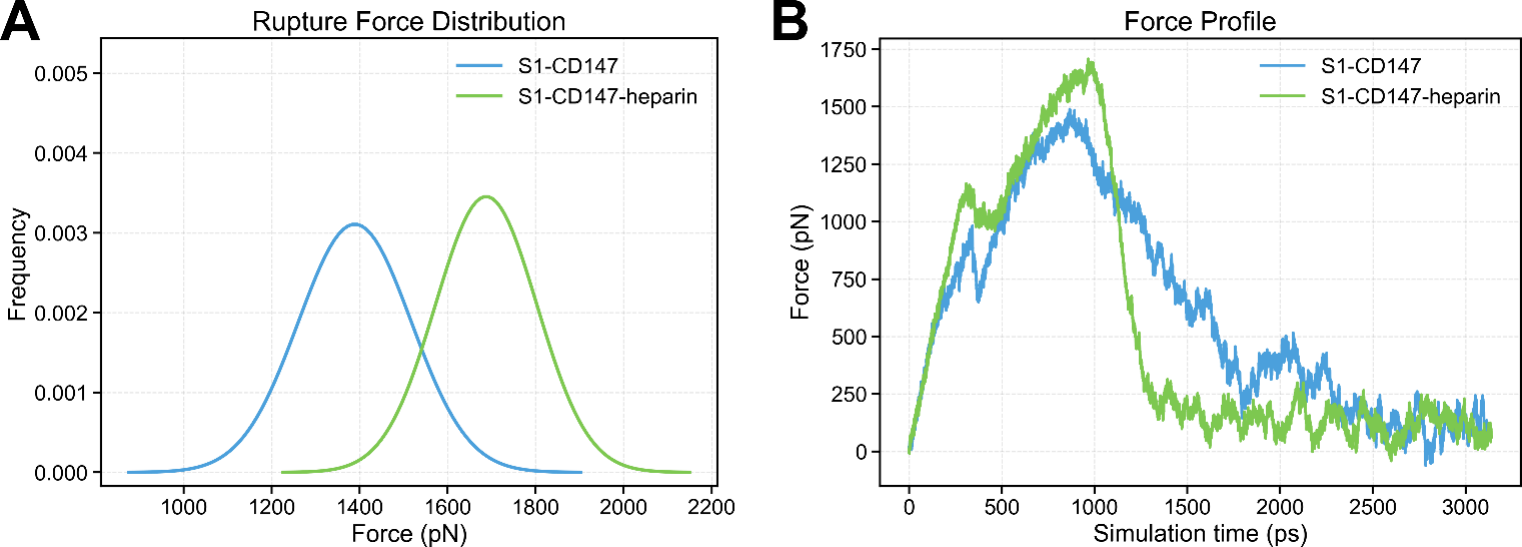
SMD of rupture forces of S1 and CD147 Complex with and without the presence of heparin. Typical force profile during SMD simulation for the unbinding process of S1-CD147 with and without the presence of heparin (A); and the average rupture force distribution and Gaussian fitting profile (B).

## Discussion

The interaction between the SARS-CoV-2 spike protein (SP) and the transmembrane glycoprotein CD147 plays a critical role in facilitating COVID-19 virus infection, yet the binding characteristics remain incompletely understood. This study provides a comprehensive investigation into the binding dynamics of CD147 and SP under various conditions, offering valuable insights into their molecular interaction mechanisms.

The ELISA assay results corroborate the affinity between the SARS-CoV-2 spike protein and CD147, reinforcing the notion that this interaction is specific and concentration-dependent. These findings support the hypothesis that CD147 may serve as a relevant coreceptor in viral entry, particularly through recognition of the S2 subunit. This supports previous findings identifying CD147 as a potential receptor for viral entry, particularly in SARS-CoV-2.^42^ The use of anti-S2 antibodies for detection proved more effective than anti-S1 antibodies, likely due to steric hindrance preventing S1 epitope recognition upon receptor binding. This aligns with studies showing that conformational changes in S1 shield its epitopes,^42^ limiting antibody detection. The small error bars indicate consistency, while the BSA blocking control minimized nonspecific interactions, confirming the specificity of SP-CD147 binding. The optimized 10 nM CD147 and 250 nM SP concentrations provided strong, reliable signals, which were defined as optimal concentrations and used in the remaining experiments. These findings reinforce the role of CD147 in viral pathogenesis and suggest anti-S2 antibodies as a superior tool for detecting SP-CD147 interactions, improving assay sensitivity across a broad concentration range. However, the ELISA method employed in this study provides a basic measure of SP-CD147 interaction but lacks the sensitivity of techniques such as QCM or AFM. While a slight, nonsignificant increase in OD was observed with higher heparin concentrations, this may indicate a weak interaction; nonetheless, definitive conclusions cannot be drawn without more sensitive and quantitative assays. Additionally, it is possible that heparin-binding sites on CD147 were randomly masked during immobilization on the 96-well plate, thereby reducing the detectable effect of heparin in this assay. Unlike QCM or AFM, the ELISA setup did not incorporate a molecular spacer between CD147 and the plate surface, which may have restricted the flexibility and orientation of CD147, thereby limiting exposure of its heparin-binding domains.

By optimizing contact durations in SMFS experiments, we established a balance between maximizing detectable binding events and minimizing nonspecific interactions. The intermediate contact time (0.5 s) was found to offer the most reliable mechanical insights into the spike-receptor interactions. The data suggest that the SARS-CoV-2 spike protein can effectively target both CD147 and ACE2. Despite this fact, these two receptors showed no significant difference in adhesion force, supporting the idea that CD147 may serve as an alternative biological target, in line with previous work on CD147 as a potential alternative target.^7,19^ Because there was a difference in binding strength between ACE2 and CD147 mean adhesion, this was further studied through the SP-coated tip contact time at 0, 0.5, and 1.0 s dwelling time levels. The dynamic nature of protein interaction likely accounts for this increase. The dynamic nature of protein interaction likely accounts for this increase in adhesion forces, as this process can induce changes in the structural conformation and the binding interface. Thus, tip-contact time was taken into account as an optimization variable in our experiments to characterize the interaction aside from the kinetic differences in the different binding interfaces. Previous work has also demonstrated that ACE2 reaches a binding maximum in dwelling times greater than 0 s, supporting the tip contact time observations in this study. Therefore, 0.5 s tip contact time was selected for the remaining SFMS experiments.

Temperature emerged as a modulating factor in receptor binding, with elevated conditions (fever-like temperatures) selectively diminishing the SP-ACE2 interaction while enhancing SP-CD147 binding. This divergence suggests distinct thermal stabilities and binding kinetics between the two receptor engagements. Temperature-dependent behavior demonstrates that elevated temperatures weaken the binding strength of SP to ACE2, likely due to increased molecular flexibility and faster bond dissociation rates. These findings align with the general principles of biomolecular interactions and provide a detailed biophysical characterization of the ACE2-SP complex. However, CD147 exhibits a stronger interaction at elevated temperatures than ACE2. These findings suggest that the SARS-CoV-2 virus may have adapted to enhance its infectivity via CD147 in different environmental conditions, including hot climates. While different host cell factors can influence the mutagenesis rate, several mutations have been identified that are preserved across different viral strains, dramatically increasing the virus’s infectivity over time. Among these, the D61mutation 4G was identified as one of the most persistent mutations in various viral strains.^43^ This mutation is proposed to provide more flexibility and accommodate SP conformational changes that favor the interaction with the biological target ACE2. Likely, the modification has been linked to higher viral loads and potentially greater transmissibility.

A study by Lim et al. in 2021 showed that SP can display more open conformations at 37 °C.^44^ They observed that at higher temperatures, like 60 °C, the SP protein can experience greater plasticity, expanding and increasing height in real-time.^44^ Although the SFMS experiment at 40 °C showed a decrease in adhesion forces, which might suggest a rearrangement in the binding interface. Contrary to predicted seasonal fluctuations, unusual infection surges were recorded in the Summer of 2022, coinciding with heatwave occurrences.^45^ Lian et al. revealed that heat waves had a notable effect on the number of cases reported globally, with an overall increase of 78%.^45^ European countries, such as France and Italy, reported 115.0% and 181.2% higher infection incidence rates, respectively, while other continents, including Asia, Oceania, and the Americas, experienced a mean 121.5% increase.^45^ Moreover, African countries like Tunisia and Ethiopia had a 298.4% increase in the number of cases.^45^

This observation was also investigated in another study by Herder et al., where a 3D Air–Liquid-Interphase cell culture model of the respiratory epithelium was used to determine the effect of elevated temperatures on the SARS-CoV-2 virus infectivity and replicability.^48^ To further evaluate whether the temperature plays a role in combination or independently from the immune system response, they quantified the expression of several crucial immune response genes that play a role in the virus infection primary response, such as interleukins and interferon-based cytokines. Researchers found that temperature conditions can modulate the SARS-CoV-2 virus replication independently from robust canonical interferon-mediated responses in febrile conditions. Lower temperatures exhibited similar transcriptomic activity to those with comparable cytokine expression levels but were associated with higher viral loads in the infected tissues.^48^ Even though it was concluded that elevated temperature decreased the transcription rate of virus genomic RNA in infected tissues, the infection rate was not statistically different. Furthermore, the comparable infectivity of the virus at elevated temperatures in this study supports the observations made by Wang et al., Lin et al., and our work.^3,38,48,51^ While it has been shown that higher temperatures can be detrimental to the virus,^48–50^ other studies have shown that the SARS-CoV-2 spike protein remains thermally stable in molecular dynamic simulations at elevated temperatures up to 50 °C.^51^ These studies highlight the intricacy of the virus infection mechanism, as well as the impact of environmental variables such as temperature. Although these unexpected trends suggest that the virus is rapidly evolving to withstand extreme environmental conditions, the underlying molecular mechanism remains to be further investigated. It may be possible that the virus has evolved to interact with alternative biological targets, such as the CD147 receptor, in an attempt to adapt to harsher environmental conditions. Although biological systems exhibit highly dynamic and multifactorial interactions among biomolecules, simplified models such as temperature-dependent binding force measurements offer a controlled platform to dissect specific interaction parameters. While our study does not capture the full metabolic complexity, the observed temperature effects provide foundational insight into thermally modulated binding behavior. This approach is commonly used to approximate binding affinities and kinetic profiles under varying energetic conditions.^46,47^ The relevance of such measurements lies in their ability to reflect key physicochemical principles that also operate *in vivo*, such as the enthalpic and entropic components of molecular recognition. These insights contribute to our understanding of how environmental conditions, including fever-range temperatures or local tissue heating, may influence molecular interactions within biological systems.

Quantitative binding studies using QCM indicated a preferential and stronger association of full-length spike protein with heparin compared to the S2 subunit. This differential binding likely reflects the contribution of electrostatic interactions involving the highly charged regions of the full spike and may implicate heparin as a modulator of spike-mediated processes. Clinical observations reported the role of heparin for COVID-19 patients with coagulopathy, while heparin interacts directly with viral spike protein, thereby exerting inhibitory effects and partially impairing its binding to host cell receptors.^14,15^ Liu J. et al. proposed that heparan sulfate may bind to SARS-COV-2 SP and block viral attachment or entry.^48^ A previous study emphasized the complex interaction of heparin and SP and demonstrated its ability to bind different regions of viral protein.^41^ Our QCM results align with this study by confirming the binding between SP and heparin. Potential contributing factors to the observed mass increase may include differences in binding affinity, stoichiometry, or secondary interactions. Notably, further structural or mutational studies would be necessary to conclusively localize binding sites. Furthermore, they suggest that heparin may reduce the SP binding potential and could hinder the virus’s ability to interact with the host cell’s target receptor. Specifically, heparin binding to the S1 subunit from SP may mask or block critical sites required for viral entry, thus acting as an inhibitor.^20,49^ The increased availability of heparin-binding sites on the S1 subunit may be attributed to its higher surface charge density. Even though the COVID-19 virus shows an overall negatively charged surface at neutral pH, there are still multiple patches of both positive and negative charge in the pH range from 5.0 to 8.0, where viruses are stable.^50^ Perhaps, the patches of the positive charge of the virus can be blocked by the negatively charged heparin. These are likely composed of lysine- and arginine-rich patches, which, under physiological conditions, can interact with the negatively charged sulfate groups of heparins.^20^ However, because both the structure and glycosylation patterns greatly influence the effective interactions with the biological target, the ACE2 receptor may have a more prevalent interaction with SP. Certain glycans not only serve as an immunogenic shield but also have been shown to affect the spatial arrangement of SP, thereby facilitating the open accessibility of the receptor binding domain.^51–53^ These open conformations could allow smaller molecules such as heparin to access and bind to SP surface areas with positively charged characteristics. The S2 subunit is involved in the membrane fusion process and is essential for viral entry following receptor binding. Although the molecular weight of the S2 subunit (60 kDa) is only slightly lower than that of the S1 subunit (76.9 kDa), the QCM experiments showed a significantly smaller mass change when heparin interacted with the S2 subunit compared to the S1 subunit. This suggests that heparin has less access to the binding sites on the S2 than the S1 subunit. Nevertheless, such interaction of heparin with both subunits highlights its multivalency as an anticoagulant and a potential modulator for viral-host interaction. Therefore, the effects of heparin on viral infectivity should be further investigated.

The introduction of heparin notably enhanced the unbinding force between SP and CD147, as revealed through SMFS. This observation points to a possible stabilizing or bridging function of heparin within the interaction interface, potentially altering the conformational landscape or accessibility of binding sites. The increase in force distribution variation further supports the notion that heparin modifies the interaction dynamics between these molecules. It has already been reported that both ACE2 and heparin can independently bind to the spike protein in vitro, suggesting that heparin can act as a scaffold to form a ternary complex, thereby influencing viral attachment and entry mechanisms.^54^ Consistently, our findings indicate that heparin also acts as a mediator in the binding process, strengthening the association between SP and CD147.

A surprising outcome of this study was the effect of heparin on the CD147-SP interaction. Contrary to its anticipated role as a binding inhibitor, heparin enhanced the binding strength. As the heparin concentration increases, the enhancement of the interaction between SP and CD147 becomes more evident. This suggests that heparin may act as a scaffold that promotes this interaction, rather than inhibiting it. This response may occur because, at certain concentrations, heparin could bridge the interactions between SP and CD147, making the binding more effective. Further characterization of the heparin-binding properties could potentially help develop more comprehensive treatments for COVID-19.

Computational modeling underscored a cooperative interaction framework, in which heparin simultaneously engages both the spike protein and CD147 without directly overlapping their binding sites. Enhanced binding energies and increased interface stabilization suggest that heparin facilitates a multivalent binding environment conducive to receptor engagement. Consistent with SMFS experimental results, the enhanced unbinding force obtained by simulations in the presence of heparin indicates a stronger interaction between S1 and CD147. The observed increase in mass may reflect additional or stronger binding interactions between heparin and the spike protein subunits, potentially contributing to a more stable complex under mechanical stress; however, further studies are required to determine the exact nature and location of these interactions. Notably, the binding energy exhibits significant dependence on the specific binding site. Helal et al. reported that a more significant binding energy was observed when CD147 bound to the bottom of S1, which contrasts with our findings.^55^ Our simulation only focused on the interaction between CD147 and the top part of S1, excluding any involvement from the S2 subunit. It is essential to acknowledge that while the binding energies calculated using the MM-GBSA approach provide valuable insights into the relative trends among various systems, they tend to yield values substantially higher than experimental measurements. However, these calculations facilitate a meaningful comparison of the different interaction strengths and their corresponding stabilities. For a more accurate representation of binding energies, it is necessary to employ alternative methods that are computationally more demanding but capable of providing absolute binding free energy values. This unexpected result underscores the complexity of molecular interactions in biological systems and suggests that the role of heparin in modulating CD147-SP binding warrants further investigation.

A previous study reported that “No evidence for basigin/CD147 as a direct SARS-CoV-2 spike binding receptor.”^13^ while others claimed that SARS-CoV-2 spike binds directly to CD147.^12,42,55^ Our results proved that even though the binding of SP to CD147 is weaker than to ACE2, its binding is enhanced at specific conditions, such as in the presence of heparin or at fever-like temperature. This observation is fundamental in understanding the role of glycosaminoglycans heparin in viral entry mechanisms, as it suggests that heparin may enhance the interaction between viral proteins and host receptors. The implications of this interaction could extend to potential therapeutic strategies, where modulation of heparin levels might influence viral attachment and entry into host cells.

The observed enhancement of spike protein binding to CD147 in the presence of heparin suggests that heparin may function as a molecular bridge or stabilizer, facilitating this interaction. While our results demonstrate an apparent increase in binding affinity under these conditions, the study shows several limitations. The specificity of this effect on CD147 has not been systematically tested against a broader range of proteins. Heparin, characterized by its high negative charge density, has the potential to bind nonspecifically to proteins enriched in basic or positively charged amino acids.^24,56^ However, in our experiments, this enhancement was not observed with unrelated control proteins such as BSA, indicating a degree of selectivity in the heparin-mediated SP-CD147 interaction. Nonetheless, further studies are necessary to determine whether this effect is unique to CD147 or if heparin may also enhance SP interactions with other host receptors or surface proteins. Understanding this broader context will be essential to clarify the role of heparin as a potential cofactor in viral entry mechanisms. In addition, while SMFS enabled us to quantify the unbinding forces between SP and CD147 with and without heparin, it did not yield direct kinetic parameters such as dissociation constants (KD) or association rates. These values typically require complementary biophysical techniques such as dynamic force spectroscopy, surface plasmon resonance (SPR), or isothermal titration calorimetry (ITC). Although we partially addressed this gap through molecular dynamics simulations and binding free energy calculations, which supported the experimental trends, future work incorporating kinetic measurements would provide a more comprehensive understanding of the interaction mechanisms and binding affinities. Furthermore, while this study focuses on SP-CD147 interactions, additional analyses of SP-ACE2 and SP-Heparin/ACE2 interactions are underway and will be reported separately. The last limitation is the absence of direct SP-Heparin interaction measurements as a nonspecificity control. Immobilizing heparin on surfaces requires chemical modifications that alter its native structure and charge, potentially introducing artifacts. To avoid such confounding effects and preserve its biological integrity, we did not include this control. To summarize our findings, the effects of fever temperature and heparin on the binding dynamics of the SARS-CoV-2 spike protein with its primary receptors, ACE2 and CD147, are visualized in Figure 7.

**Figure 7:**
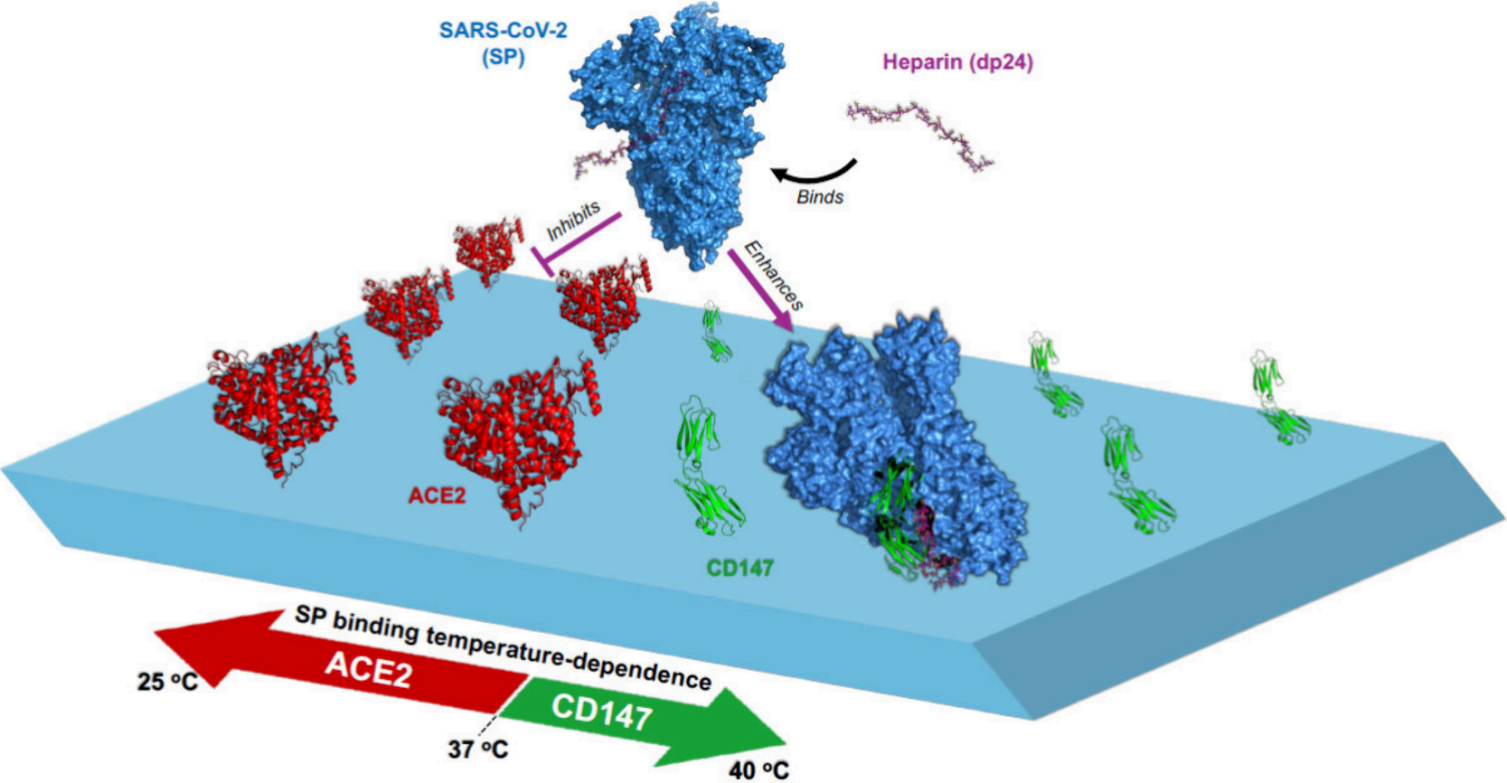
Proposed model illustrates the SARS-CoV-2 binding bivalency to biological target receptors modulated by environmental factors. In this model, the SP preferably binds to ACE2 (dark red) at lower temperatures (∼25 °C), while binding to CD147 (green) is enhanced at elevated temperatures (>37 °C). The presence of heparin (dp24, purple) may cause SP-ACE2 inhibition but enhances the SP-CD147 interaction, suggesting a temperature- and cofactor-dependent modulation of receptor binding.

It illustrates how environmental conditions modulate these interactions and emphasizes that elevated temperatures (∼40 °C) weaken SP-ACE2 interactions, reducing viral attachment stability due to increased molecular flexibility and accelerated bond dissociation rates. In contrast, SP-CD147 interactions are strengthened under fever conditions, suggesting that the virus may utilize CD147 more effectively when ACE2 binding becomes less favorable. Additionally, the unexpected role of heparin in these interactions is depicted. Rather than inhibiting viral attachment, heparin selectively enhances SP-CD147 binding while preventing SP-ACE2 interactions. This dual effect is attributed to heparin’s ability to act as a stabilizing bridge between SP and CD147, reinforcing their interaction, particularly under elevated temperature conditions. The visualization underscores the potential shift in receptor utilization by SARS-CoV-2 under fever conditions. The depiction highlights the auxiliary role of CD147 in viral entry, especially when ACE2-mediated pathways are impaired. Furthermore, the illustration serves as a basis for considering therapeutic strategies that target CD147 or modulate heparin interactions to influence viral entry dynamics. This visualization aids in conceptualizing the interplay between temperature, heparin, and receptor binding, reinforcing the study’s broader implications.

Understanding the detailed binding characteristics of CD147 and SP can guide the development of targeted therapeutic strategies to disrupt this interaction, potentially mitigating SARS-CoV-2 infectivity. The unexpected role of heparin as a binding enhancer also highlights the need for careful evaluation of therapeutic agents and their multifaceted effects in biological contexts.

## Conclusion

This study provides a detailed characterization of the interaction between CD147 and the SARS-CoV-2 spike protein (SP), highlighting the effects of key experimental factors such as contact time, temperature, and the presence of heparin. Using a combination of biophysical techniques and molecular simulations, we confirmed the binding of CD147 to SP and uncovered the nuanced dynamics of this interaction. Prolonged contact times were found to strengthen the binding of SP. However, this study revealed that elevated temperatures can increase the adhesion of SP to CD147, as the interaction seems to weaken with the primary target ACE2. Unexpectedly, heparin enhanced the CD147-SP binding rather than inhibiting it, likely due to its simultaneous binding to both molecules. Molecular simulations complemented these findings by offering atomic-level insights into the interaction mechanisms. These results broaden our understanding of the CD147-SP interaction and may significantly impact therapeutic strategies targeting this pathway. The findings underscore the importance of considering environmental and molecular factors when designing inhibitors or therapeutic agents. This work contributes to a growing body of knowledge aimed at mitigating SARS-CoV-2 infectivity and improving treatment strategies for COVID-19.

## Supplementary Material



## Notes

Binding of CD147 to SP detected by using anti-S1 antibodies, Control experiments to determine the range of the nonspecific adhesion forces with different samples for analysis, Effect of heparin of different concentrations on the binding of CD147 to SP, Root-mean-square deviation (RMSD) of three independent runs of the most stable complex during a 200 ns MD simulation, Molecular interaction analysis of the spike protein S1 and CD147 complex during the final nanosecond of MD simulation. (PDF)
