# Supplementary material for: Molecular Insights into the Interaction between CD147 and the SARS-CoV‑2 Spike Protein
Source: ACS Omega. 2025 Aug 5;10(32):36025–40. doi: 10.1021/acsomega.5c03562 (PMC12368639; doi:10.1021/acsomega.5c03562)
Supplement: Supplementary file 1 [file ao5c03562_si_001.pdf]

Supplementary Information

Molecular Insights into the Interaction Between CD147 and the SARS-CoV-2 Spike Protein

Milton S. Gonzalez-Serrano,[1,2†] Li-Yu Chen,[1†] Hemi Desai,[3] Thi Huynh Ho,[4,5] Doris Heinrich,[1,6] Thuat Thanh Trinh,[7*] and Thi-Huong Nguyen,[1,6*]

[1]*Institute for Bioprocessing and Analytical Measurement Techniques, Heiligenstadt, Germany*

[2] *Chemical and Biomolecular Engineering Department, Ohio State University, Columbus, Ohio, United States of America*

[3]*Hochschule Anhalt University of Applied Sciences, Köthen, Germany*

[4]*Laboratory for Computational Physics, Institute for Computational Science and Artificial Intelligence, Van Lang University, Ho Chi Minh City, Vietnam*

[5]*Faculty of Mechanical - Electrical and Computer Engineering, School of Technology, Van Lang University, Ho Chi Minh City, Vietnam*

[6]*Faculty of Mathematics and Natural Sciences, Technische Universität Ilmenau, 98694 Ilmenau, Germany*

[7]*Porelab, Department of Chemistry, Norwegian University of Science and Technology, Høgskoleringen 5, Trondheim 7491, Norway*

E-mail: thi-huong.nguyen@iba-heiligenstadt.de, thuat.trinh@ntnu.no

Figure S1: Binding of CD147 to SP detected by using anti-S1 antibodies.

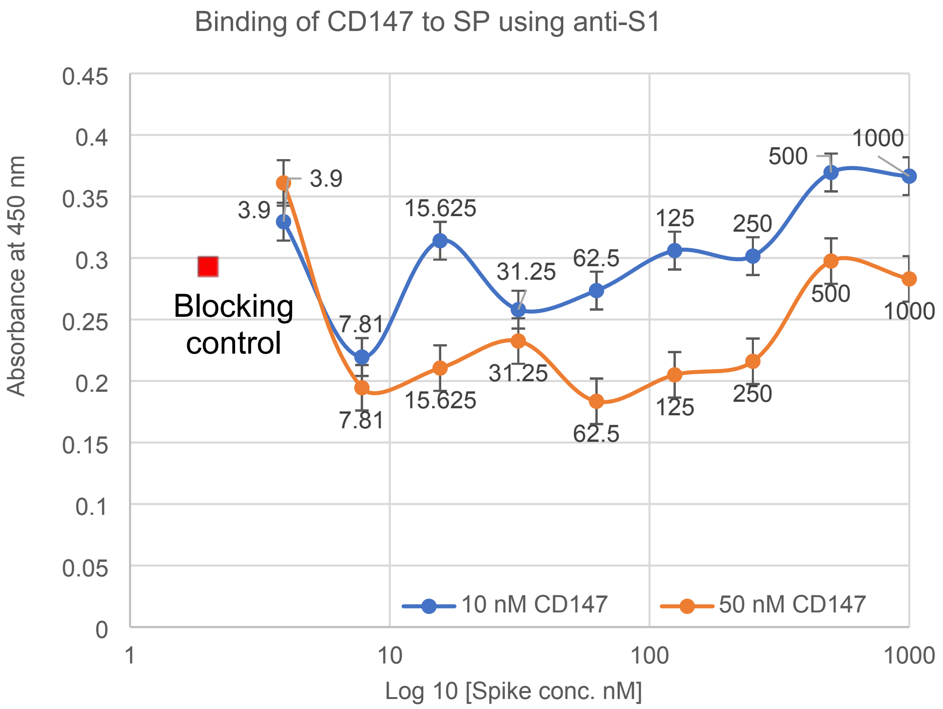

CD147 and SP bind weakly using anti-S1 as the primary antibody where the SP value ranges from 3.9 nM to 1000 nM, and the value of CD147 is 10 nM (blue) and 50 nM (orange). The orange square indicates the blocking control (0.285, ± 0.026), the addition of 7.5% BSA to the wells followed by SP, and primary and secondary antibodies. Error bars indicate the mean standard deviation for two experiments (n=2). Absorbance was measured at 450 nm.

Figure S2. Control experiments to determine the range of the non-specific adhesion forces with different samples for analysis.

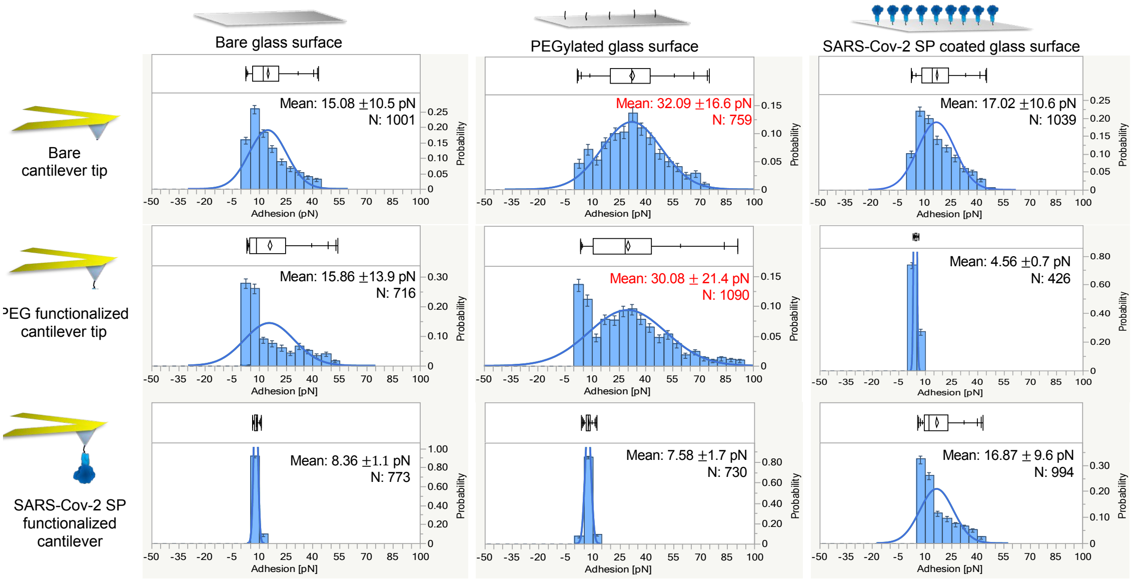

Figure S3. Effect of heparin of different concentrations on the binding of CD147 to SP.

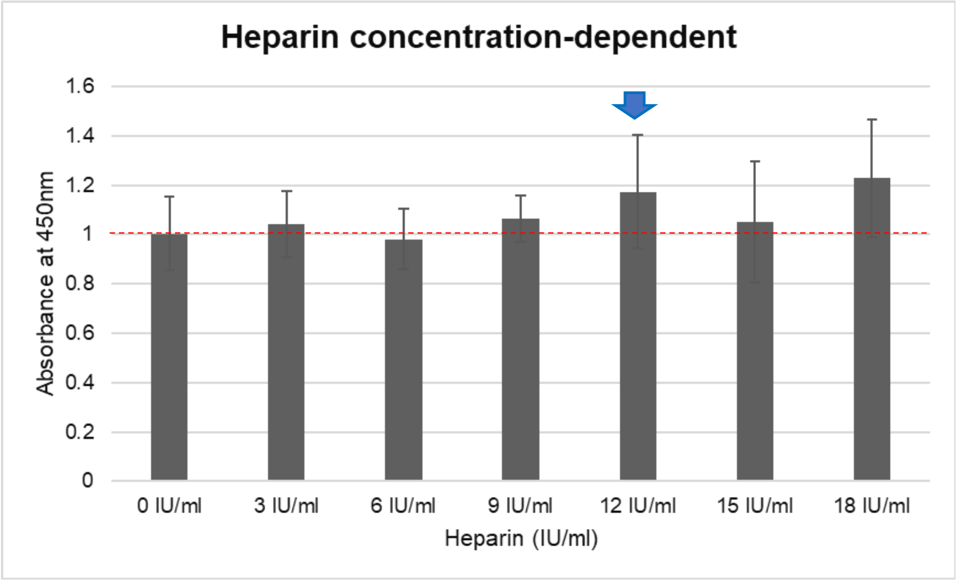

SP and CD147 binding with different units of unfractionated heparin, where 0 IU/ml indicates no heparin addition which is considered as baseline (red). At concentrations ≥ 12IU/mL (arrow), a slight increase in OD is observed but lack of significant compared to ‘no heparin’(P = 0.808). Error bars indicate the mean standard deviation. (n=3)

Figure S4. Root-mean-square deviation (RMSD) of three independent runs of the most stable complex during a 200 ns MD simulation.

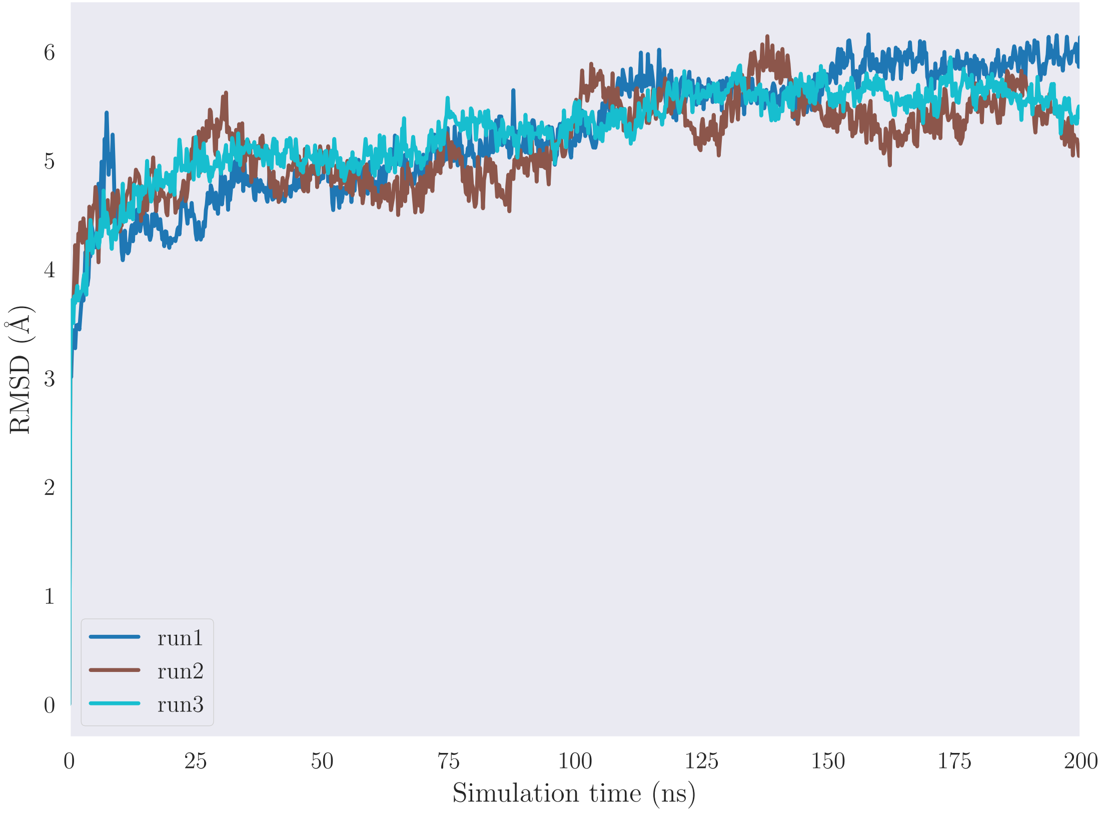

This complex is the most stable one among 3 docking complexes (see Table 1 in the main text).

Figure S5. Molecular interaction analysis of the spike protein S1 and CD147 complex during the final nanosecond of MD simulation.

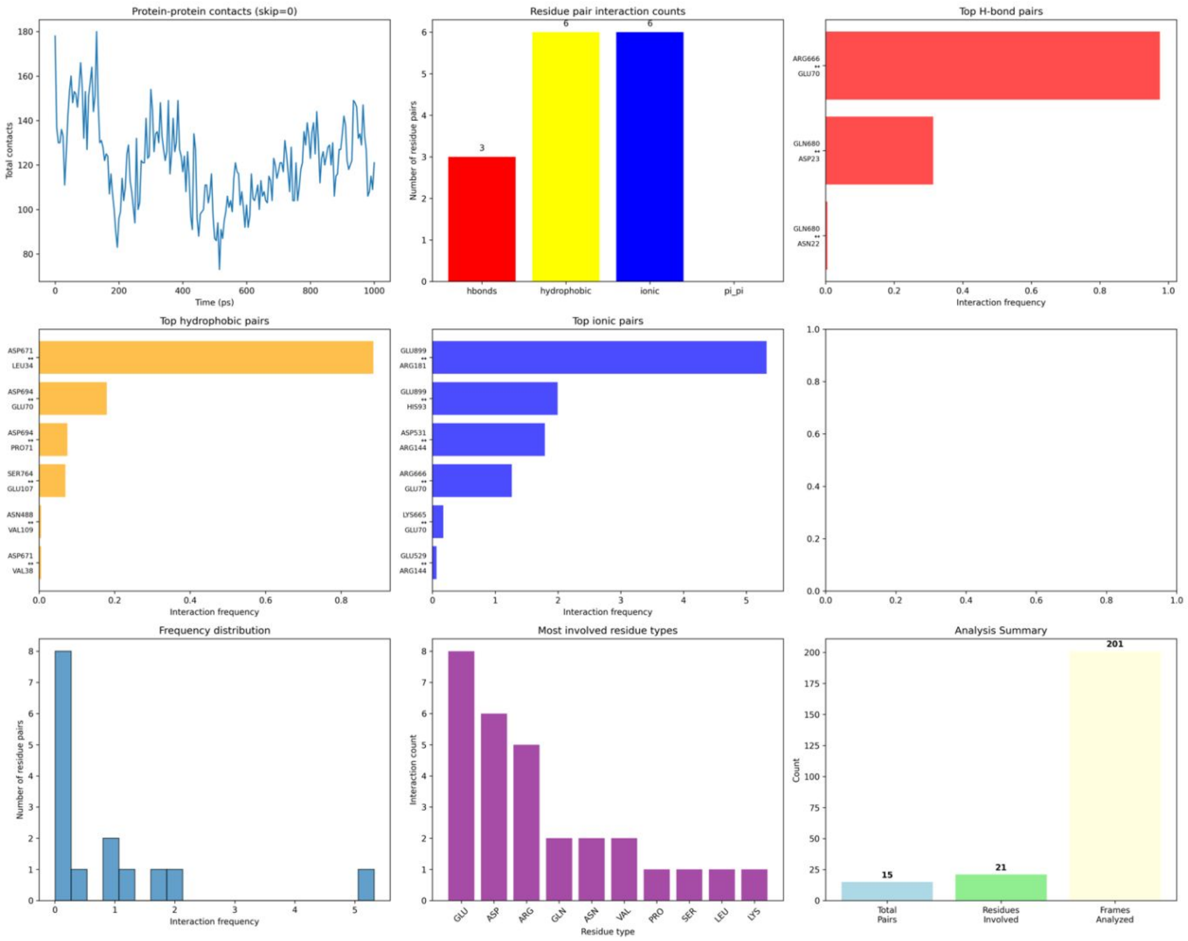

Figure S5 depicts the protein-protein interaction analysis between the spike protein (S1) and CD147 during the last nanosecond of the molecular dynamics (MD) simulation. This figure demonstrates that both proteins exhibit strong interactions, with a majority of residue pairs participating in hydrophobic, hydrogen bond, and ionic interactions. Notably, there are no π-π stacking interactions observed between S1 and CD147. Figure S2 also provides a comprehensive list of various top interaction pairs between the S1 and CD147.
